# Unscheduled changes in pre-clinical stroke model housing contributes to variance in physiological and behavioural data outcomes: A post hoc analysis

**DOI:** 10.1177/23982128241238934

**Published:** 2024-03-20

**Authors:** Aisling McFall, Delyth Graham, Stuart A. Nicklin, Lorraine M. Work

**Affiliations:** School of Cardiovascular & Metabolic Health, University of Glasgow, Glasgow, UK

**Keywords:** Animal housing, pre-clinical stroke, corncob, physiology

## Abstract

Ischaemic stroke presents a significant problem worldwide with no neuroprotective drugs available. Many of the failures in the search for neuroprotectants are attributed to failure to translate from pre-clinical models to humans, which has been combatted with rigorous pre-clinical stroke research guidelines. Here, we present post hoc analysis of a pre-clinical stroke trial, conducted using intraluminal filament transient middle cerebral artery occlusion in the stroke-prone spontaneously hypertensive rat, whereby unscheduled changes were implemented in the animal housing facility. These changes severely impacted body weight post-stroke resulting in a change from the typical body weight of 90.6% of pre-surgery weight post-stroke, to on average 80.5% of pre-surgery weight post-stroke. The changes also appeared to impact post-stroke blood pressure, with an increase from 215.4 to 240.3 mmHg between housing groups, and functional outcome post-stroke, with a 38% increased latency to contact in the sticky label test. These data highlight the importance of tightly controlled housing conditions when using physiological or behavioural measurements as a primary outcome.

## Introduction

Ischaemic stroke remains a significant burden as the third leading cause of death and disability combined worldwide (Feigin et al., 2021). Although advances have been made with mechanical thrombectomy representing a significantly improved treatment option for stroke (Nogueira et al., 2018), no neuroprotective drugs have yet been identified to help repair or protect the brain following ischaemia-reperfusion injury. The failure of many previously trialled neuroprotective strategies has largely been attributed to limitations in pre-clinical stroke study design and, as a result a series of guidelines have been produced and updated by the stroke community, namely the stroke therapy academic industry roundtable (STAIR) recommendations (Fisher et al., 2009), the RIGOR guidelines (Lapchak et al., 2013) and the IMPROVE guidelines (Percie du Sert et al., 2017). These recommendations aim to improve the quality of pre-clinical studies so that drugs or therapies are more likely to succeed in clinical trials where the population is more heterogeneous and displays multi-morbidities. In particular, the IMPROVE guidelines incorporate considerations for animal housing, environment and bedding, which had not been considered previously (Percie du Sert et al., 2017).

With these guidelines in mind, a therapeutic intervention study was designed using the stroke prone spontaneously hypertensive rat (SHRSP), which is a comorbid animal model with chronic hypertension and resulting end-organ damage (Kato et al., 2015), and has worsened outcome following stroke compared to its normotensive reference strain, the Wistar Kyoto rat (McGill et al., 2005) or the less hypertensive spontaneously hypertensive rat (SHR; Maguire et al., 2004). Despite design of a robust study in line with published guidelines, the study was terminated early with underpowered group sizes due to severity limits of the project licence being reached, which subsequently led to inconclusive results regarding the intervention.

Importantly, however, uncontrollable and unscheduled variables were also introduced during the study, with changes made to the bedding and cage type used within the animal facility. A surprising drop in weight of some animals was then observed, despite otherwise relatively normal health and behaviour, and it was proposed that the bedding or cage changes may have impacted outcome following stroke. Therefore, data were analysed post hoc to assess if these changes had impacted physiological and stroke outcome measures.

## Methods

### Animals

Male SHRSP (*n* = 12, 16–23 weeks old, 270–310 g at time of surgery), bred ‘in-house’ were used in this study. Animals had *ad libitum* access to rodent chow (RM1 expanded diet, SDS, England) and untreated tap water. All studies were carried out in accordance with the Animals Scientific Procedures Act 1986 and approved by the University of Glasgow’s Animal Welfare & Ethical Review Board.

### Housing

Animals were kept in either standard polycarbonate RB3 cages (240 × 120 × 400 mm base + additional 80 mm of cage top; original cages) or GP2000P cages (340 × 200 × 430 mm base + additional 45 mm of cage top; new cages) in groups of two to three, with standard wood shavings (Andersons, Bo’ness, Scotland) or corncob bedding (DBM ltd, Broxburn, Scotland), which was subject to weekly changes. Following surgery, animals were singly housed in standard polycarbonate RB3 cages.

### Blood pressure measurements

Systolic blood pressure readings were recorded using non-invasive tail-cuff plethysmography in the four channel rat platform BP-2000 Blood Pressure Analysis System™ (Visitech Systems, Bioseb, UK) using a 10 s pulse analysis period and a maximum pressure of 250 mmHg. Blood pressure measurements were recorded following 3 days of prior acclimatisation to the apparatus. Four baseline measurements were performed before surgery and three measurements post-surgery to improve the reliability of the readings.

### Surgical procedures

Four days prior to stroke surgeries, animals received transcranial burr hole surgery to improve survival and recovery (Ord et al., 2012). The stroke model utilised was transient middle cerebral artery occlusion (tMCAO) using an intraluminal filament. Briefly, a silicone-coated monofilament (Doccol Corporation, USA) was advanced through the left carotid artery to block the left MCA. The filament was tied in place for 35 min before removal to allow reperfusion. Animals were recovered, singly housed and maintained for 10 days post-tMCAO.

### Functional outcome measures

Functional outcome was assessed at baseline and 3, 7 and 10 days post-tMCAO, using a 30-point neurological score, with a battery of nine tests to assess mobility, limb function and general health (Ord et al., 2013), the tapered beam test where footfaults of the limbs on the affected side of the body were counted and expressed as a percentage of total footsteps taken (Ord et al., 2013), and the sticky label test (Bouet et al., 2009), where the animal’s latency to contact a label on its affected forepaw was measured. Further detail is in the Supplemental Materials. Animals were also weighed at the time of testing.

### Infarct measurement

Animals were humanely killed 10 days post-tMCAO by transcardiac perfusion with phosphate-buffered saline followed by 4% paraformaldehyde and brains were processed by an alcohol and xylene gradient and embedded in paraffin wax. Infarcts were measured across five coronal levels spanning the region of the MCA by first staining with haemotoxylin and eosin and then identifying healthy and infarcted cortical tissue (Ord et al., 2013). The infarcted area was transcribed onto images from the rat brain atlas and measured using ImageJ software. Further details are in the Supplemental Materials.

### Data analysis

For functional and weight analysis, the area under the curve (AUC) was calculated for each animal and t-test with Welch’s correction performed on the AUC values. Additional analyses were performed with two-way repeated measures analysis of variance (ANOVA) on GraphPad Prism 9.2 to assess differences at each time point (Bonferroni’s multiple comparisons test) or to assess difference from baseline (Dunnett’s multiple comparisons test). Blood pressure was analysed with a two-way ANOVA with Bonferroni’s multiple comparisons test while infarct volume was assessed by t-test with Welch’s correction. Retrospective power calculations were also performed and these values are indicated in the results section for significant (*p* < 0.05) results.

## Results

**Figure 1.**
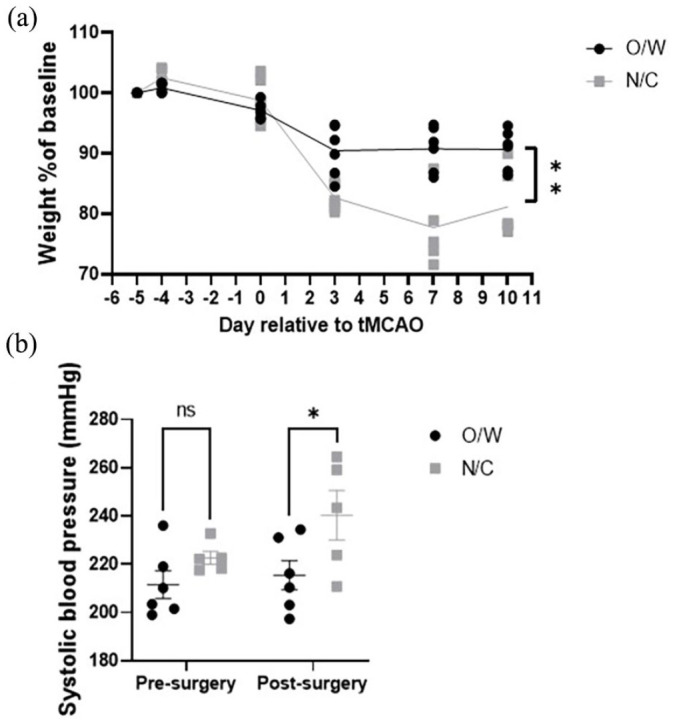
Effect of cage type and bedding on physiological measures post-stroke. (a) Weight of SHRSP presented as % of baseline weight (day –5, one day before burrhole surgery) for rats housed in original cages before and after surgery with wood shavings as bedding (O/W; black circles) or for rats housed in new, larger cages (prior to surgery only) with corncob as bedding (N/C; grey squares). Each animal is represented by a single data point and line represents the mean for each group. **p = 0.0017 t-test with Welch’s correction performed on AUC values for each animal. (b) Systolic blood pressure measurements of O/W (black circles) and N/C rats (grey squares) measured using tail cuff plethysmography pre-surgery (average of four occasions prior to burrhole) and post-surgery (average of three occasions after tMCAO). *p = 0.03, ns: not significant p = 0.5, two-way ANOVA with Bonferroni’s correction for multiple comparisons, O/W versus N/C.

The first six animals of the study were housed and recovered with cage bedding of standard wood shavings and in original cages. During the study, bedding in the facility was switched to corncob and the cage type used to house stock animals prior to surgery was changed to new cages (total *n* = 6 in this cage/bedding combination). These new cages were larger in size allowing more room for exploration and enrichment; however, they were unavailable for housing post-surgery. Observation of weight data demonstrated that those animals with the corncob bedding and experiencing the larger new cage type prior to surgery before being switched to smaller original cages post- surgery (new cage & corncob group, N/C), had significantly lower body weights across the course of the study compared to those on standard wood shavings and in the original cage type both before and after surgery (original cage + wood shavings group, O/W; see Figure 1(a); *p* = 0.0017**, 93% power). Further analysis of the data demonstrated that body weight did not differ between groups prior to tMCAO surgery (day 0; day –5 to day 0, *p* = 0.1) but only after tMCAO surgery where O/W animals were on average 90.6% of their pre-surgery weight while N/C animals were on average 80.5% of their pre-surgery weight (day 3 to day 10, *p* = 0.0008***).

N/C animals also showed significantly higher blood pressure post-surgery (240.3 ± 22.9 mmHg) compared to those housed in the O/W condition (215.4 ± 14.9 mmHg) but there was no significant effect of surgery overall (O/W versus N/C pre-surgery *p* = 0.5, post-surgery *p* = 0.03*, 27% power, O/W versus N/C overall *p* = 0.014*, pre-surgery versus post-surgery overall *p* = 0.12; see Figure 1(b)).

**Figure 2.**
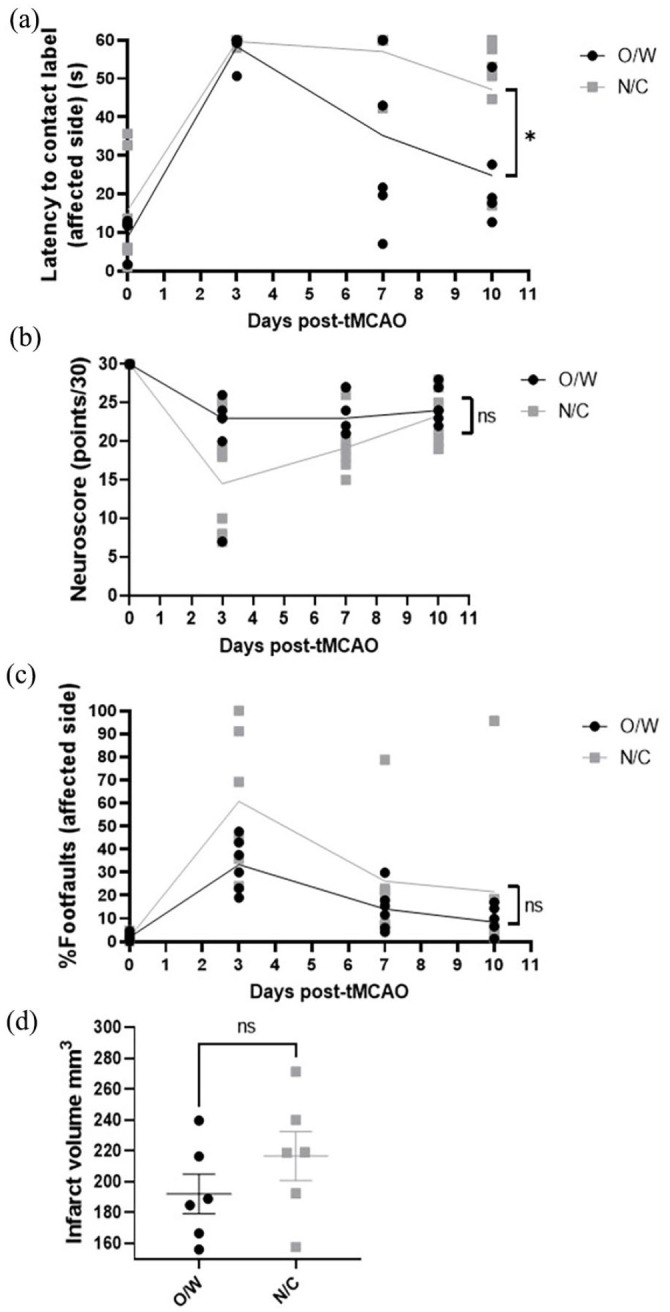
Effect of cage type and bedding on functional and histological measures post-stroke. Results of the (a) sticky label test, (b) neurological score test battery and (c) tapered beam test employed at baseline and 3, 7 and 10 days post-tMCAO to measure functional outcome. Each animal is represented by a single data point: black circles represent rats housed in original cages before and after surgery with wood shavings as bedding (O/W), grey squares represent rats housed in new, larger cages (prior to surgery only) with corncob as bedding (N/C). The line represents the mean (a and c) or median (b) for each group. *p = 0.017 t-test O/W versus N/C with Welch’s correction performed on AUC values for each animal, ns: not significant. (d) Infarct volume measured across five coronal levels at day 10 post-tMCAO, ns: not significant *p* = 0.26.

In terms of stroke outcome, N/C animals displayed poorer performance in the sticky label task assessing tactile response overall with N/C animals taking an average of 54.6 s to contact the label on days 3–10, whereas O/W took an average of 39.4 s (see Figure 2(a); *p* = 0.017**, 61% power) and O/W rats showed recovery in task performance by 10 days post-stroke while N/C rats retained a deficit (baseline versus day 10 *p* = 0.19, N/C baseline versus day 10 *p* = 0.04*). There was no significant effect of the bedding or cage type on neurological score (*p* = 0.11), tapered beam test performance (*p* = 0.15), or infarct volume (*p* = 0.26) post-stroke; however, there was a trend towards a greater deficit in both neuroscore and tapered beam test performance in the N/C group at day 3 post-stroke (day 3 neuroscore *p* = 0.07, day 3 %footfaults *p* = 0.1; see Figure 2(b)–(d)).

## Discussion

These data demonstrate an important retrospective finding within an *in vivo* pre-clinical stroke study showing a significant negative impact of unscheduled changes to animal unit facilities on outcome post-tMCAO surgery, supporting the requirement for tightly controlled environmental conditions in these studies.

The change of bedding from wood shavings to corncob may have affected weight due to previous reports suggesting negative hormonal effects due to corncob bedding in Sprague-Dawley (SD) rats (Kang et al., 2015; Markaverich et al., 2002) and the potential negative impact endocrine disruptions can have on stroke recovery (Franceschini et al., 2001). In addition, however, mixing of the corncob grains with the soft diet was observed, which may have resulted in accidental consumption. Ingestion of corncob bedding can lead to alterations in the gut microbiota in SD rats (Le Leu et al., 2015), and there is evidence for microbiota dysbiosis having a negative impact on stroke outcome (Singh et al., 2016), or lead to reduction in feed conversion in WT mice (the weight gained for food consumed; Ambery et al., 2014), both of which could account for the reduction in weight observed in this study with corncob bedding after stroke.

Corncob bedding has also previously been shown to reduce the amount of time SD rats spend in slow-wave sleep, which can disrupt animal physiology and neurological function and may be a sign of elevated stress (Leys et al., 2012). Studies in both rats (Gao et al., 2010; Zunzunegui et al., 2011) and humans (Joa et al., 2017; Sonmez and Karasel, 2019) have suggested that sleep disruptions are detrimental to stroke recovery and poorer sleep has also been associated with hypertension (Lo et al., 2018).

Therefore, one could speculate that if the corncob bedding disturbed sleep, this could increase blood pressure and/or result in poorer recovery post-stroke in terms of weight loss. In contrast to these results, corncob bedding has previously been shown to attenuate salt-induced hypertension in Dahl salt-sensitive rats (Dasinger et al., 2022) and to impair vascular reactivity to phenylephrine in some mice strains (Sveeggen et al., 2023). Nevertheless, this post hoc analysis adds to the evidence of bedding being a confounding factor for studies utilising physiological outcome measures.

The other key change in facilities was the introduction of larger stock cages for the rats, which allowed groups of three to four rats to be housed more comfortably with more space for enrichment such as additional cardboard tubes or toys. This is in line with NC3Rs guidelines for rodent housing (www.nc3rs.org.uk/3rs-resources/housing-and-husbandry/rodents) and the IMPROVE guidelines for refinement of *in vivo* stroke procedures (Percie du Sert et al., 2017). Unfortunately, however, these cages were unavailable for housing post-surgery where rats were singly housed in smaller cages. Despite recommendations for animals not to be singly housed when on procedure (Percie du Sert et al., 2017), it was not possible to group rats in this study due to welfare concerns if a rat experienced post-tMCAO fitting.

Rat housing has been extensively studied with cage type and contents being the most investigated housing refinement (Neville et al., 2023). Previous studies have shown that environmental enrichment (EE; where housing is enhanced with additional objects to interact with and the size of the cage may also be increased) reduces stress in laboratory animals, and improves depressive-live behaviours and performance in learning and memory tests (Simpson and Kelly, 2011). Direct assessment of the influence of cage size suggests that Wistar rats show a preference for larger cages (Patterson-Kane, 2002) and that male Wistar rats housed individually, in a cage similar in size to the O/W cages in this study, showed less stress in terms of corticosterone levels compared to when housed with four or more other male rats (Brown and Grunberg, 1995) suggesting that male Wistar rats prefer larger less-crowded cages.

Importantly, there is also a large volume of evidence suggesting EE improves outcome following experimental stroke (McDonald et al., 2018). Interestingly, EE generally reduces body weight (Simpson and Kelly, 2011) and does not appear to affect post-stroke weight loss in pre-clinical studies (Gresita et al., 2022; Risedal et al., 2002) including a study by Risedal et al. where SHR were moved from larger cages with cagemates pre-stroke to singly housed smaller cages post-stroke (Risedal et al., 2002). Therefore, it is possible that the cage size had less impact on the post-stroke weight loss and this effect was more due to the bedding change, however, without an appropriately controlled study no definitive conclusion can be made.

The downsizing of cage size post-stroke may, however, have detrimentally impacted the sticky label outcome measure as EE has been shown to improve motor recovery post-stroke (McDonald et al., 2018) and in particular in the Risedal et al. study, the rotarod performance of rats placed in smaller, singly housed caged post-stroke was very poor (Risedal et al., 2002). It is worth noting that only the time to contact the sticky label was significantly impacted, which is an assessment of tactile asymmetry and sensorimotor dysfunction. Meanwhile, the performance in the tapered beam or neurological assessment, which are more motor function-based failed to reach statistical significance, although trends towards a greater deficit in the N/C group were observed in these motor-based tests at the earliest time point of day 3 post-stroke. This may be due to a greater sensitivity of the sticky label test to detect subtle dysfunction, indeed this type of test is regarded as a sensitive test in the stroke field (Ruan and Yao, 2020), or potentially differing underlying mechanisms whereby early motor deficits in the N/C group are resolved while tactile deficits become progressively worsened.

Cage size may also have impacted blood pressure whereby no difference in blood pressure in small versus large caging conditions has been reported (Brauner et al., 2010) while others report increased blood pressure in rats in singly housed non-enriched cages, but only in high anxiety rats (Ravenelle et al., 2014). Therefore, perhaps an additional stressor, such as anxiety, stroke surgery or chronic hypertension, is required to see the blood pressure effects of housing.

Interestingly, while these changes appeared to have had an impact on body weight, blood pressure and one of three behavioural measurements, there was no impact on infarct volume. These findings suggest that the stroke insult did not vary between animals and was not affected by the housing changes. Therefore, this highlights the importance of avoiding environmental changes for any studies that use physiological or behavioural measurements as the primary outcome measure.

Overall, there is an indication that cage or bedding changes may have had negative impact on outcome post-stroke due to retrospective observations. These results should be considered with this limitation in mind as it is possible that observations were due to another unknown cause or entirely coincidental. Group sizes are small and, with the exception of the weight loss effect, the variation between animals means that statistical tests lack appropriate power. Therefore, further hypothesis-driven welfare studies are required to confirm a causative effect of bedding and/or housing for these observations; for example, housing naïve and post-stroke rats in various bedding and housing combinations, or switching naïve normotensive rats and SHRSP between the cage and bedding combinations and back again. It is also worth noting that these data reflect male rats only due to the technical difficulty regarding filament size required for female rats. Therefore, it would be important to include female animals in future studies to ascertain if any of these effects are sex specific. Nevertheless, despite these limitations, these data emphasise the need for highly controlled environments in experimental stroke studies, as changes in external factors, such as bedding or cages, may result in poorer outcomes or highly variable results.

## Supplemental Material

sj-jpg-2-bna-10.1177_23982128241238934 – Supplemental material for Unscheduled changes in pre-clinical stroke model housing contributes to variance in physiological and behavioural data outcomes: A post hoc analysis Supplemental material, sj-jpg-2-bna-10.1177_23982128241238934 for Unscheduled changes in pre-clinical stroke model housing contributes to variance in physiological and behavioural data outcomes: A post hoc analysis by Aisling McFall, Delyth Graham, Stuart A. Nicklin and Lorraine M. Work in Brain and Neuroscience Advances

sj-pdf-1-bna-10.1177_23982128241238934 – Supplemental material for Unscheduled changes in pre-clinical stroke model housing contributes to variance in physiological and behavioural data outcomes: A post hoc analysis Supplemental material, sj-pdf-1-bna-10.1177_23982128241238934 for Unscheduled changes in pre-clinical stroke model housing contributes to variance in physiological and behavioural data outcomes: A post hoc analysis by Aisling McFall, Delyth Graham, Stuart A. Nicklin and Lorraine M. Work in Brain and Neuroscience Advances
