# Supplementary figures and images for: Unscheduled changes in pre-clinical stroke model housing contributes to variance in physiological and behavioural data outcomes: A post hoc analysis
Source: Brain Neurosci Adv. 2024 Mar 20;8:23982128241238934. doi: 10.1177/23982128241238934 (PMC10956152; doi:10.1177/23982128241238934)

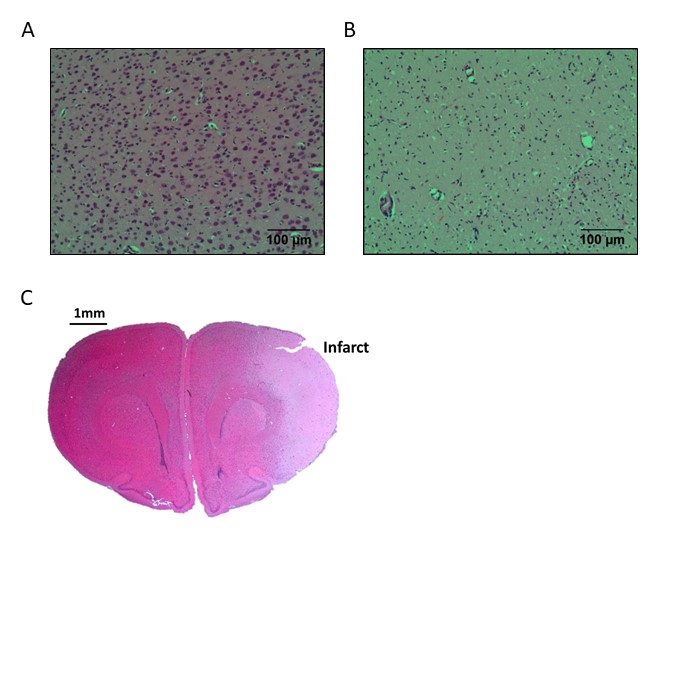

Supplement: sj-jpg-2-bna-10.1177_23982128241238934 – Supplemental material for Unscheduled changes in pre-clinical stroke model housing contributes to variance in physiological and behavioural data outcomes: A post hoc analysis [file sj-jpg-2-bna-10.1177_23982128241238934.jpg]
