# Supplementary material for: Unscheduled changes in pre-clinical stroke model housing contributes to variance in physiological and behavioural data outcomes: A post hoc analysis
Source: Brain Neurosci Adv. 2024 Mar 20;8:23982128241238934. doi: 10.1177/23982128241238934 (PMC10956152; doi:10.1177/23982128241238934)
Supplement: sj-pdf-1-bna-10.1177_23982128241238934 – Supplemental material for Unscheduled changes in pre-clinical stroke model housing contributes to variance in physiological and behavioural data outcomes: A post hoc analysis [file sj-pdf-1-bna-10.1177_23982128241238934.pdf]

Supplemental Methods file for:

Unscheduled changes in pre-clinical stroke model housing contributes to variance in physiological and behavioural data outcomes- a post-hoc analysis

Authors

Aisling McFall*, Delyth Graham, Stuart A Nicklin, Lorraine M Work

School of Cardiovascular and Metabolic Health, University of Glasgow.

*corresponding author

In Brain and Neuroscience Advances

<u>Functional outcome measures</u>

Functional outcome was assessed, at baseline and 3, 7 and 10 days post-tMCAO, using the following three tests.

1. Neurological score

A battery of nine tests were used to assess mobility, limb function and general health, described below, to give a 30-point score (the lower the score the worse the deficit). Scoring is detailed in Table S1.

Paw Placement: The animal was held at the edge of the bench and each leg was gently pulled over the edge and the animal's ability to retract the paw was scored. If it failed to retract the paw within 5 seconds it was counted as unsuccessful.

Horizontal bar: The animal was held by the base of its tail so that its nose was level with a thin (5mm diameter) horizontal rod, held in place with a retort stand, and its front paws were allowed to contact the rod. The tail was slowly lowered to allow the animal to hang and the opportunity to raise its hindlimbs onto the bar. This was performed twice and the score for the best attempt was recorded.

Rotation: The animal was placed on the bench, held by the base of the tail and rotated in a clockwise and anti-clockwise direction. The ability of the animal to move against to rotation direction was scored.

Visual forepaw reaching: The animal was held by the base of the tail so that its vibrissae were just touching the bench edge. The animal was scored on its ability to reach and *place* its front paws on the bench. If the animal reached but did not successfully place the paw it was scored as unsuccessful.

Grip strength: The animal was held by the base of its tail over a wire cage top so that its front paws could reach the cage. It was then pulled backwards and was scored on the grip strength of the affected (right side) paw.

Response to vibrissae touch: The animal was placed on a bench and allowed to wander freely. Approaching from behind, a finger or pen was brushed against the whiskers on each side successively and the animal's response scored.

Circling: The animal was placed on the floor and allowed to roam freely. Then scored on its ability to move normally or the extent of its circling behaviour.

Mobility: The animal's willingness to move and mobility was observed while roaming freely on the floor for circling assessment.

General condition: The animal was weighed and its general appearance was assessed.

Table S1 Criteria for 30-point Neurological Score

**Paw Placement**	Score: **1** for each successful paw placement **(max = 4)**
**Horizontal Bar**	Score: **3** if both hindlimbs raised onto the bar<br>**2** if one hindlimb raised onto the bar<br>**1** if the animal hangs from the bar<br>**0** if the animal falls
**Rotation**	Score: **1** for each side **(max = 2)**
**Visual Forepaw Reaching**	Score: **1** for each successful paw placement **(max = 2)**
**Grip Strength**	Score: **3** for normal strength<br>**2** for good but weakened strength<br>**1** for weak strength<br>**0** for no grip
**Response to vibrissae touch**	Score: **3** equal response on both sides<br>**2** weakened response on affected side<br>**1** no response on affected side<br>**0** no response on either side
**Circling**	Score: **5** if the animal does not circle<br>**4** if the animal tends to one side<br>**3** for large circles (> 50 cm radius)<br>**2** for medium circles (15-50 cm radius)<br>**1** for small circles (> 15 cm radius)<br>**0** for spinning or refusing to move
**Mobility**	Score: **4** for normal mobility<br>**3** for very active<br>**2** for lively<br>**1** for unsteady<br>**0** if reluctant to move
**General Condition**	Score: **4** if normal/good (shiny coat, no weight loss)<br>**3** if good, but some weight loss<br>**2** if good, but weight loss + has secretions (eyes/nose) OR dirty coat<br>**1** if good but weight loss + has secretions + dirty/unkempt coat<br>**0** if animal has lost weight + has secretions + dirty,unkempt coat + hunched/pinched posture

2. Tapered beam test

Limb function was also assessed using the tapered beam test (Schallert, Woodlee, and Fleming 2002). Each animal was trained to traverse a 130cm tapered beam to reach its home cage and then tested three times at each time-point. The test was recorded to allow for the number of footsteps and number of footfalls to be counted. The percentage of footfalls on each side was then calculated for each animal and averaged from the three attempts.

3. Sticky label test

Tactile response and asymmetrical response was assessed using the sticky label test (Bouet et al. 2009). A small (8mm diameter) sticky dot label was placed on the underside of each of the animal's forepaws. The animal was then placed in an open-topped box and video-recorded for up to 120 seconds or until both labels had been removed; this was repeated three times. The videos were replayed and the latency (seconds) to contact the label was recorded and averaged for the three attempts. If the animal did not contact the label within 60 seconds, the trial was brought to an end and it was given a maximal score of 60 sec for contact time.

Infarct analysis

Infarcts were measured across five coronal levels spanning the region of the MCA by first staining with haemotoxylin and eosin and then identifying healthy and infarcted cortical tissue (Ord et al., 2013). Healthy cortical tissue was defined as having a homogenously stained neuropil and regular shaped, rounded nuclei (Figure S1A) while infarcted cortical tissue had lighter stained and vacuolated neuropil with pyknotic (small and triangular) shaped nuclei (Figure S1B). Infarcts were transcribed onto images from the rat brain atlas (Paxinos and Watson 2007), to correct for increased volume due to cerebral oedema, before being measured using ImageJ software. Total infarct volume was calculated using an area under the curve calculation on GraphPad Prism 4.0.

Figure legend:

Figure S1 Infarct identification by H&E staining. Representative images of the SHRSP brain 3 days post-tMCAO stained with H&E showing **(A)** healthy cortex with homogenously stained neuropil and regular shaped, rounded nuclei and **(B)** infarcted cortex with paler staining, vacuolated neuropil and pyknotic (small and triangular) shaped nuclei. **(C)** A representation of the gross appearance of an H&E stained SHRSP brain section is also shown.
